# The Biodistribution of ^99m^Tc Labelled Equine Peripheral Blood Mesenchymal Stem Cells in Healthy and Chronic Gingivostomatitis Cats

**DOI:** 10.3390/vetsci13080727

**Published:** 2026-07-23

**Authors:** Charlotte Beerts, Yves Debosschere, Liesa Tack, Glenn Pauwelyn, Stephanie Carlier, Eva Depuydt, Jimmy H. Saunders, Kathelijne Peremans, Jan H. Spaas

**Affiliations:** 1 UniLaSalle, Université d’Artois, IDEALISS, ULR 7519, 76130 Mont-Saint-Aignan, France; 2 Boehringer Ingelheim Veterinary Medicine Belgium NV, Noorwegenstraat 4, 9940 Evergem, Belgium; lieasatack@hotmail.com (L.T.); glenn_pauwelyn@hotmail.com (G.P.); stephaniecarlier@outlook.com (S.C.); eva.depuydt.94@gmail.com (E.D.); janspaas@hotmail.com (J.H.S.); 3 Department of Morphology, Imaging, Orthopedics, Rehabilitation and Nutrition of Domestic Animals, Faculty of Veterinary Medicine, Ghent University, Salisburylaan 133, 9820 Merelbeke, Belgium; yves@vetdent.be (Y.D.); jimmy.saunders@ugent.be (J.H.S.); kathelijne.peremans@ugent.be (K.P.)

**Keywords:** biodistribution, chronic gingivostomatitis, equine peripheral blood, feline, mesenchymal stem cells, xenogeneic

## Abstract

Feline chronic gingivostomatitis (FCGS) is a chronic inflammatory disease of the oral mucosa. Mesenchymal stem cell (MSC) treatments have been investigated as a potential treatment for this condition; however, the biodistribution profile of MSCs remains poorly understood. The goal of this study was to assess the relative pharmacokinetics of MSCs by comparing their biodistribution in healthy cats to that in cats suffering from FCGS. Two studies were performed to evaluate the biodistribution of radiolabelled equine peripheral blood-derived mesenchymal stem cells (ePB-MSCs) following intravenous and subcutaneous administration in healthy cats (*n* = 3) and in cats with FCGS (*n* = 4). Total body scintigraphic scans were obtained using a gamma camera. Free ^99m^Technetium accumulation was primarily observed in the heart, lungs, stomach, bladder, thyroid, and salivary glands. Following intravenous administration of radiolabelled ePB-MSCs, increased radiopharmaceutical uptake (IRU) was mainly detected in the lungs, liver, kidneys, and bladder. After subcutaneous administration, IRU was predominantly observed at the injection site. In cats affected by FCGS, a higher radiotracer signal was observed in the rostral oral region following intravenous administration, with a mean 3.4-fold increase compared with healthy cats. The biodistribution pattern of radiolabelled ePB-MSCs differed from that of free ^99m^Technetium. These findings provide preliminary information regarding the biodistribution of radiolabelled ePB-MSCs in cats.

## Simple Summary

Feline chronic gingivostomatitis (FCGS) is a painful condition that causes severe inflammation in a cat’s mouth, making it difficult for them to eat, groom, and enjoy life. Traditional treatments, like tooth extractions and medications, do not work for all cats. In this study, we investigated whether stem cells derived from equine peripheral blood could be detected in inflamed oral tissues after administration to healthy cats and cats affected by FCGS. We injected these stem cells into healthy cats and into cats affected by FCGS and used special imaging techniques to track where the cells went. In cats with oral inflammation, an increased radiotracer signal was detected near the inflamed oral region following intravenous administration of labelled stem cells. This suggests that the cells may have the potential to help treat this disease by localizing to regions affected by inflammation. These findings are a first step in understanding how such treatments could offer a new option for cats suffering from this difficult condition.

## 1. Introduction

Feline chronic gingivostomatitis (FCGS) is a painful disease that often leads to a poor quality of life and reduces the life expectancy of affected cats [1,2,3,4]. The disease is characterized by severe inflammatory lesions in the oral cavity that can be erosive and/or proliferative in nature. These lesions not only affect the gingiva and alveolar region but also extend to the labio-buccal and caudal oral mucosa, typically found lateral to the palatoglossal folds [2,4,5].

The exact cause of FCGS is not fully understood, but it is believed to result from an inappropriate immune response to chronic oral antigenic stimulation [2,3,4]. Several infectious and non-infectious factors have been implicated, including viruses, bacteria, other dental diseases, and dental-plaque-associated antigens [2,4]. Dysregulation of CD8+ lymphocytes has been reported and is thought to contribute to disease pathogenesis [6]. Recent molecular studies further support the role of immune dysregulation in FCGS, demonstrating upregulation of inflammatory and immune-related pathways [7,8]. Mesenchymal stem cells are known to migrate toward sites of tissue injury and inflammation following systemic administration. This process is mediated by interactions between chemokines, growth factors, adhesion molecules, and endothelial cells within the inflammatory microenvironment [9]. Given the inflammatory microenvironment associated with FCGS, characterization of MSC biodistribution may provide insight into whether intravenously administered ePB-MSCs preferentially localize to affected oral tissues. Regardless of the causal factors, affected areas always show a chronic inflammatory involvement, characterized by lymphoplasmacytic infiltration of the mucosa and submucosa [1,4,10,11].

Treatment of FCGS is challenging due to its multifactorial nature. Current pharmacological treatment options involve the use of immunosuppressive or anti-inflammatory drugs in combination with antimicrobials [2,5]. However, the standard of care often involves partial or full-mouth tooth extraction, with success rates ranging from 70% to 80% of cases. Unfortunately, 20% to 30% of cats do not respond to these treatments [1,2,4,7].

As an alternative approach, researchers have investigated the use of autologous and allogeneic mesenchymal stem cells (MSCs) following tooth extraction. Although the exact mechanism of action is not fully understood, it has been observed that adipose-derived MSCs (ASCs) can decrease T cell proliferation and modulate their cytokine secretion. Furthermore, disease remission or substantial improvement and resolution of oral lesions were reported [1,10,12]. Transcriptomic approaches have also been explored to better characterize treatment responses in FCGS, including in cats receiving MSC therapy. Differences in gene expression profiles have been reported between MSC responders and cats responding to tooth extraction alone. In particular, the activation of pathways related to autophagy, IL-8 signaling, integrin signaling, and hepatic fibrosis has been described in MSC responders [7]. These observations suggest that molecular profiling may help to explain interindividual variability in treatment response and could contribute to the development of more-targeted therapeutic strategies. However, autologous MSC therapy remains challenging in cats because the isolation and expansion of feline MSCs can be affected by syncytial cell formation, proliferation arrest and feline foamy virus (FFV) infection, which may compromise cell manufacture and quality [13]. In addition, feline MSCs have limited proliferative capacity, potentially restricting large-scale production [14,15]. The use of non-autologous MSCs avoids tissue collection from diseased patients and allows the generation of standardized, ready-to-use cell products from carefully selected healthy donors. Equine peripheral blood-derived MSCs (ePB-MSCs) represent an attractive xenogeneic cell source because they can be obtained through minimally invasive blood collection, expanded efficiently in culture, and manufactured under standardized conditions. Furthermore, previous studies have demonstrated the safety and immunomodulatory properties of ePB-MSCs in healthy cats. These immunomodulatory properties provide the biological rationale for investigating ePB-MSCs in FCGS, a disease characterized by chronic immune-mediated oral inflammation. Moreover, xenogeneic ePB-MSCs have been shown to be well tolerated in cats while retaining their capacity to modulate immune responses, supporting their evaluation as an off-the-shelf cell therapy in this species [16]. In addition, a validated method for radiolabelling ePB-MSCs with ^99m^Tc has previously been established by our group [17], making these cells a suitable model to investigate MSC biodistribution in cats. However, further research is needed to better understand their biodistribution, mode of action, and therapeutic potential in FCGS.

We hypothesized that intravenously administered radiolabelled ePB-MSCs would exhibit a biodistribution pattern distinct from free ^99m^Tc and that inflammatory lesions associated with FCGS could influence their distribution. Therefore, the objective of this exploratory pilot study was to evaluate the biodistribution of radiolabelled ePB-MSCs following intravenous and subcutaneous administration in healthy cats and cats affected by FCGS.

## 2. Materials and Methods

### 2.1. Animals and Experiments

The different animal studies (approval number EC: 2020_001 and 2020_003) and the blood collection from the donor horses (approval number: EC_2018_002) were approved by the ethics committee of Boehringer Ingelheim Veterinary Medicine Belgium, with independent members evaluating the application. The ethics committee is approved by the Flemish government with permit LA1700607. The studies were good clinical practice compliant (VICH GL9), and all procedures were conducted according to European, national, and regional animal welfare regulations (Directive 2001/82/EC as amended, Belgian animal welfare legislation (KB 29/05/2013), Directive 2010/63/EU and EMEA/CVMP/816/00-Final). Furthermore, deontological exemption was granted by the Flemish government (DWZ/KF/20/1.15/56).

In an initial controlled study, the biodistribution of ePB-MSCs was evaluated in three healthy cats following intravenous injection in the cephalic vein, subcutaneous injection in the neck, and subcutaneous injection in the inguinal region. In a second cross-over study, the biodistribution of intravenously and subcutaneously (in the neck) administrated ePB-MSCs was evaluated in four cats suffering from FCGS. No formal power calculation was performed because no prior feline MSC biodistribution data were available. Sample size was based on feasibility and the exploratory nature of the study. The healthy cats were research animals housed at the institutional facility. The cats with FCGS were client-owned animals presented for clinical management. Diagnosis of FCGS was based on bilateral caudal stomatitis with characteristic inflammatory lesions lateral to the palatoglossal folds, and the exclusion of alternative oral inflammatory diseases as determined by the attending clinician. Information regarding previous dental extractions and ongoing medical treatments was recorded. For both studies, the experimental unit was the individual animal. Due to animal welfare considerations, during the days of the scintigraphic examinations, the cats were housed in pens next to each other so that permanent olfactorial and auditive contact between cats was possible. General physical examinations were performed before treatment administration and again at the final imaging time point (24 h post-injection) during each treatment period. The assessments included rectal temperature, respiratory rate, heart rate, mucous membrane evaluation, capillary refill time, body condition score, mentation, and hydration status. All clinical examinations were performed by the same veterinarian to ensure consistency. The owners’ informed consent was obtained for all client-owned animals prior to study start. At the completion of the study and following a final veterinary health assessment, all research cats were adopted into private homes.

### 2.2. Ethical Considerations

Subcutaneous injection sites were selected in accordance with current feline injection-site sarcoma (FISS) prevention recommendations. FISS is most commonly associated with chronic inflammatory reactions, particularly following adjuvanted vaccinations or repeated injections. In contrast, subcutaneous or intravenous administration of radioactive iodine (^131^I) has been routinely performed for the treatment of feline hyperthyroidism in large clinical cohorts without reports of increased injection-site sarcoma incidence [18]. Importantly, ^131^I involves substantially higher radiation exposure than diagnostic ^99m^Tc tracers. In the present study, ^99m^Tc was administered as a single low-dose diagnostic radionuclide with a short half-life (~6 h). Animals were clinically monitored, and no local adverse reactions were observed during the study period.

Radiation safety procedures were conducted in accordance with institutional and national regulations governing the use of radioactive materials. Preparation and handling of ^99m^Tc labelled ePB-MSCs were performed by authorized personnel at a study site accredited by the Federal Agency for Nuclear Control (FANC). Personnel used appropriate personal protective equipment, including laboratory coats, gloves, and overshoes, and radiation exposure was monitored using personal dosimeters. Manipulation of ^99m^Tc was performed behind a radiation protection shield in accordance with institutional radioprotection standard operating procedures.

### 2.3. Inclusion and Exclusion Criteria

For both studies, cats had to be at least 12 months old. For the first study, cats had to be in good general health, and for the second study, the cats had to be in good general health status apart from the target disease, based on a general physical examination on day 0 and medical history for the last 2 months. Moreover, the cats had to test negative for feline immunodeficiency virus and feline leukemia virus infection. No animals were excluded from the study.

### 2.4. Control Product Preparation

The control product (CP) was obtained by adding between 185 and 370 megabecquerel (MBq) of freshly eluted ^99m^Tc Pertechnetate (^99m^TcO4^-^), depending on radionuclide availability from a molybdenum generator (GE Health Care, Eindhoven, The Netherlands), to 1 mL of Dulbecco’s Modified Eagle low-glucose Medium (DMEM) (Life Technologies Europe BV, Halle, Belgium).

### 2.5. Collection and Culture of ePB-MSCs

As previously described by our group [19], the ePB-MSCs were manufactured according to good manufacturing practices (GMPs) on a GMP-certified site (number: BE/GMP/2018/123). MSCs were isolated from the blood of a 4-year-old healthy warmblood donor gelding (approval number: EC_2018_002) housed in a dedicated donor facility. Donor horses undergo daily clinical examinations and are screened for a broad panel of transmissible infectious diseases by Böse laboratory (Harsum, Germany) before entering the donor facility and at regular intervals thereafter. As described earlier by our group [20], a centrifugation of the blood was performed, and the buffy coat was collected for gradient centrifugation. Subsequently, the ePB-MSCs were put in culture until passage 5, and cells were characterized for viability, morphology, immunophenotype (positive expression of CD29, CD44, CD90, and CD105; negative expression of MHC II, CD45, CD79α, and a monocyte/macrophage marker), and population doubling time. These quality-control assessments were repeated at passage 10. In addition, product release was performed according to predefined GMP quality-control procedures, including sterility and endotoxin testing. The ePB-MSCs were stored at −80 °C as an intermediate cell stock. The manufacturing of the final batches was done by thawing the vials of intermediate cell stock and placing in culture until passage 10. Expansion to passage 10 was performed to obtain sufficient cell numbers for clinical application while maintaining standardized manufacturing conditions. Previous studies have demonstrated the safety and immunomodulatory properties of these ePB-MSCs in cats [16]. Next, the ePB-MSCs were trypsinized, resuspended, filtered twice through a 40 µm filter and vialed at 3 × 10^5^ cells/mL in a mixture of DMEM and 10% dimethylsulfoxide (DMSO). The vials were kept at −80 °C until their use. Transport of the ePB-MSCs was done on dry ice and was temperature monitored.

### 2.6. ^99m^Tc Labelling of the ePB-MSCs

The ^99m^Tc labelling of the ePB-MSCs was performed as recently described by our group [17]. Briefly, the stannous chloride powder (Sigma Aldrich, Saint Louis, MO, USA) was liquefied in sterile basic water. Then, for the first and the second study, respectively, 9 × 10^5^ and 6 × 10^5^ ePB-MSCs were thawed in the hand palm, transferred into culture medium (DMEM supplemented with fetal bovine serum), and centrifuged for pelleting. The cell pellet was then resuspended in saline and mixed with SnCl2 and 518 t 629 MBq of freshly eluted ^99m^TcO4^-^. Next, the preparation was incubated at room temperature before being centrifuged. The cell pellet was washed with DMEM and centrifuged again. The final cell pellet was resuspended in DMEM, and the viability of the ePB-MSCs following the labelling was determined.

### 2.7. Treatment

In the first study, each of the three healthy cats received six separate injections administered at different timepoints, with a minimum washout period of 6 days between injections. Each cat received both the control product (CP) and the ^99m^Tc labelled ePB-MSCs by three administration routes: intravenous injection in the cephalic vein, subcutaneous injection in the neck, and subcutaneous injection in the inguinal region.

In the second study, each cat with FCGS received two injections of ^99m^Tc labelled ePB-MSCs in a crossover design, consisting of one intravenous injection and one subcutaneous injection in the neck. Two cats first received the intravenous injection followed by the subcutaneous injection, whereas the administration order was reversed for the two remaining cats. A washout period of at least 6 days was respected between injections.

In both studies, cats were placed under light general anesthesia and positioned in sternal recumbency on the gamma camera before each injection. Sedation was achieved using dexmedetomidine (40 µg/kg IM), followed by induction and maintenance with propofol administered to effect. Intravenous injections were administered through a 22-gauge catheter placed in a cephalic vein, whereas subcutaneous injections were administered in the dorsal cervical or inguinal region, depending on the study protocol.

### 2.8. Imaging Protocol and Image Interpretation

The biodistribution of the CP and the investigational veterinary product (IVP) was assessed by a whole-body scan using a two-headed gamma camera, equipped with low-energy high-resolution collimators (GCA 7200 A; Toshiba, Kawasaki, Japan). A quantification of the radioactivity was obtained using the free-hand region of interest tool of a DICOM viewing software (Hermes MultiModality^TM^, Nuclear Diagnostics, Stockholm, Sweden) in different manually drawn regions of interest on the dorsal and ventral view of the whole-body scans. Regions of interest were delineated using consistent anatomical landmarks for each organ and were drawn by the same experienced nuclear medicine specialist for all examinations to ensure consistency. Radioactive uptake (counts) in these regions of interest was quantified for each time point. A geometric mean of dorsal and ventral activity was calculated for each ROI and time point to compensate for tissue attenuation.

For the first study, radiopharmaceutical uptake was expressed in % of total body for each organ at the different time points. During the second study, a point source with known radioactivity was used to calculate % injected dose (ID). Therefore, relative uptake was expressed as % of decay-corrected injected activity for each region of interest per time point. As the two studies used different quantification approaches (% total body radioactivity in study 1 and % injected dose in study 2), direct quantitative comparison between these measures was not performed.

The total body acquisitions were performed 1 h, 6 h, and 24 h following the injection of the radioactive compound for both studies. Care was taken when the cat was re-positioned on the table to avoid too much spatial deviation on the scans following the first scan.

For study 2, the biodistribution in the buccal region following intravenous injection was measured by comparing the background-corrected radiopharmaceutical uptake in the rostral region of the head with the background-corrected uptake in the total head. The results were expressed in mean counts per pixel.

## 3. Results

### 3.1. Animals

For the first study, two males and one female were included. In the second study, two males and two females were enrolled. All animals were included in the Intention-to-Treat population, which was used for clinical tolerance and biodistribution evaluation. As there were no major protocol deviations, no data were excluded.

### 3.2. Control Product Preparation

The activity of the CP injected into each cat in study 1 can be found in Table 1.

### 3.3. Labelling Efficiency, Post-Labelling Viability, and Injected Dose

In the first study, the labelling efficiency ranged from 63% to 88%, and the cell viability ranged from 53% to 84% for all MSCs samples. The injected samples contained 430,000 to 885,000 MSCs and had a ^99m^Tc dose between 216.82 and 306.36MBq (Table 2).

In the second study, a labelling efficiency between 51% and 89% and a cell viability between 77% and 88% were observed for all MSC samples. The number of cells amounted at least 200,000, and ^99m^Tc doses between 176.49 and 269.73 MBq were injected (Table 3).

### 3.4. Clinical Tolerance

The parameters rectal temperature, respiratory rate, heart rate, mucosal membranes, capillary refill time, body condition score, mentation, and hydration were within the physiological range for all animals at all time points of observation for both studies. No abnormal general clinical signs were observed, and no adverse events or suspected adverse drug reactions were observed during the studies.

### 3.5. Biodistribution

**Figure 1:**
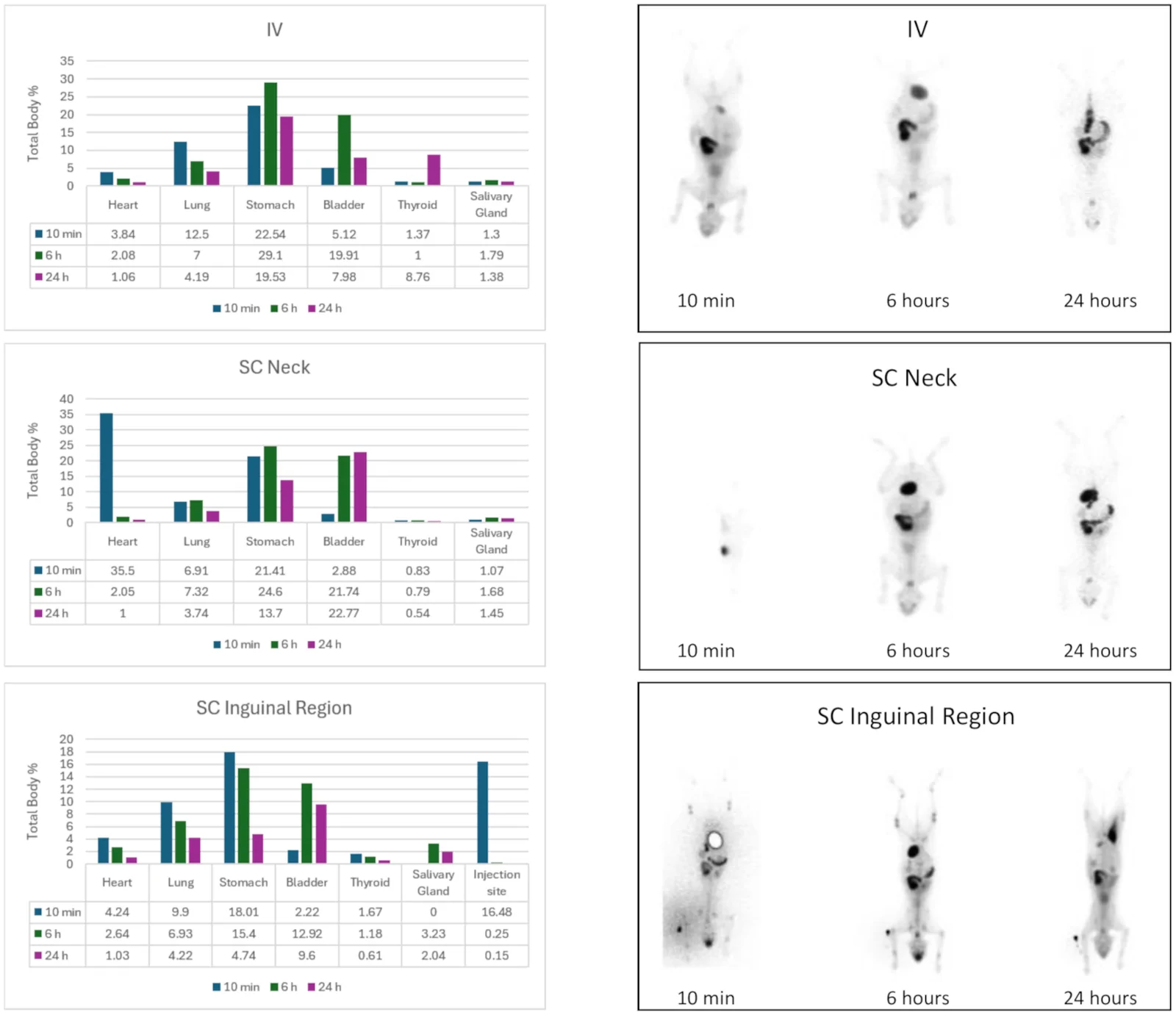
Distribution of ^99m^Tc in healthy cats. Average percentage of total body radioactivity observed in the different organs (**left**) and measured radioactivity (**right**) following the control product injections in healthy cats. IV: intravenous, SC: subcutaneous.

Following IV injection of the CP, an accumulation of free ^99m^Tc was observed in the heart, lung, stomach, thyroid, salivary glands, and bladder of all three cats. In one cat, increased radiopharmaceutical uptake (IRU) was also observed in the liver, and in another cat, IRU was seen in the intestines. The highest radiopharmaceutical uptake of free ^99m^Tc was measured in the stomach, with a progressive increase to 29.10% of the total body 6 h following the IV injection. At 24 h following the injection, IRU in the stomach amounted to 19.53% of the total body accumulation. Subcutaneous injection of the CP in the neck led to an IRU of free ^99m^Tc in the following organs: heart, lung, stomach, bladder, thyroid, and salivary glands. In one cat, a very high IRU was seen in the heart 10 min after injection. For all three cats, an IRU that augmented over time to an average of 24.6% of the accumulation in the total body at 6 h was seen in the stomach. Finally, after subcutaneous injection of free ^99m^Tc in the inguinal region, IRU was seen in the heart, lung, stomach, bladder, thyroid, and salivary glands. The highest IRU was measured in the stomach 10 min after the injection (Figure 1).

**Figure 2:**
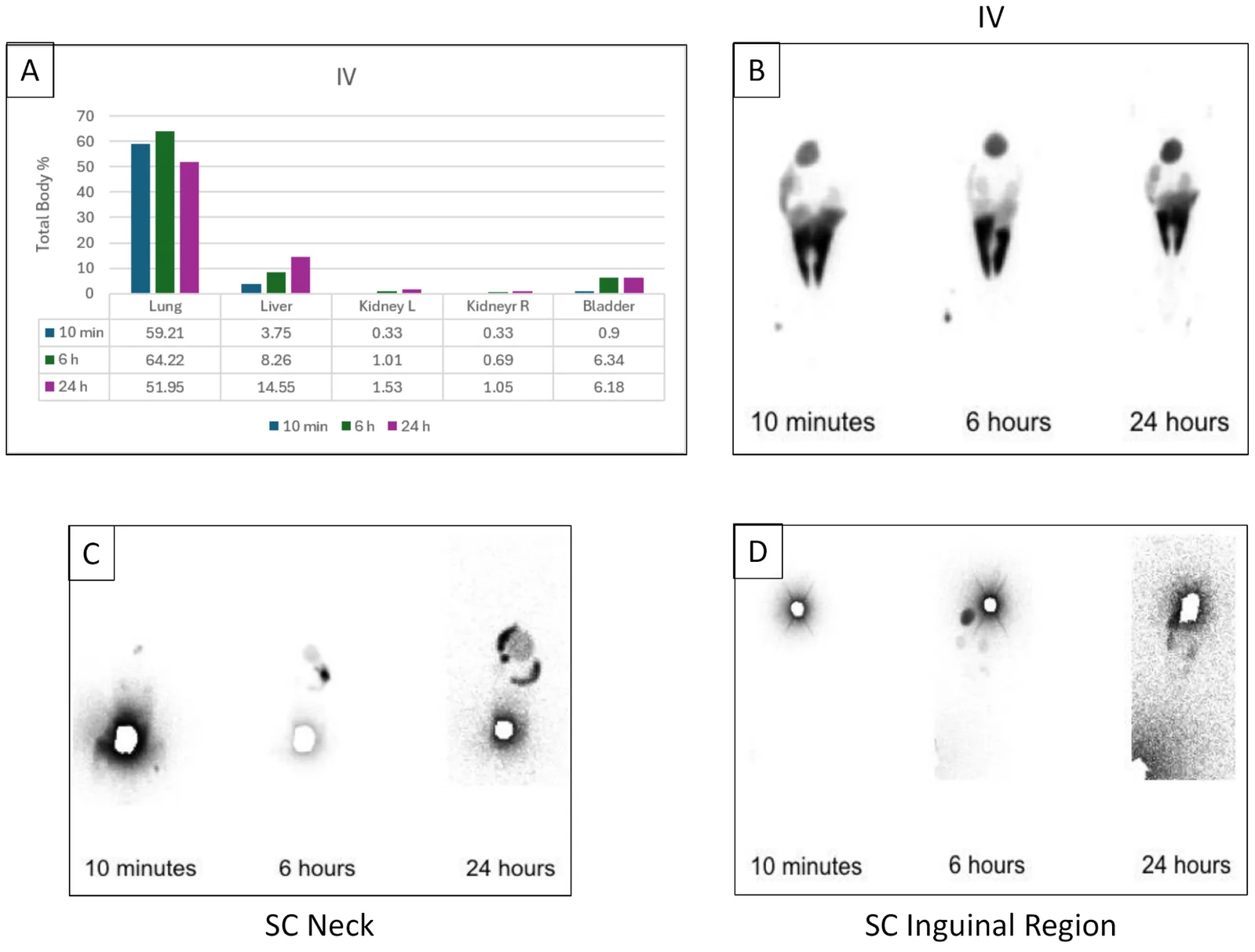
Distribution of ^99m^Tc labelled MSCs in healthy cats. Average percentage of total body observed in the different organs (**A**) and measured radioactivity at different time points (10 min, 6 h and 24 h) following intravenous (IV) injection (**B**) and subcutaneous (SC) injection of the ^99m^Tc labelled ePB-MSCs in the neck (**C**) and in the inguinal region (**D**) of healthy cats.

Intravenous injection of the ^99m^Tc labelled ePB-MSCs in healthy cats led to increased radiopharmaceutical uptake (IRU) in the lungs, liver, kidneys, and bladder. An initial pulmonary accumulation was observed at the early imaging time points, with the highest IRU detected in the lungs and increasing until 6 h post-injection. The radiopharmaceutical uptake in the liver and kidneys progressively increased following the injection. The radiopharmaceutical uptake in the bladder increased until 6 h post-injection (Figure 2).

**Figure 3:**
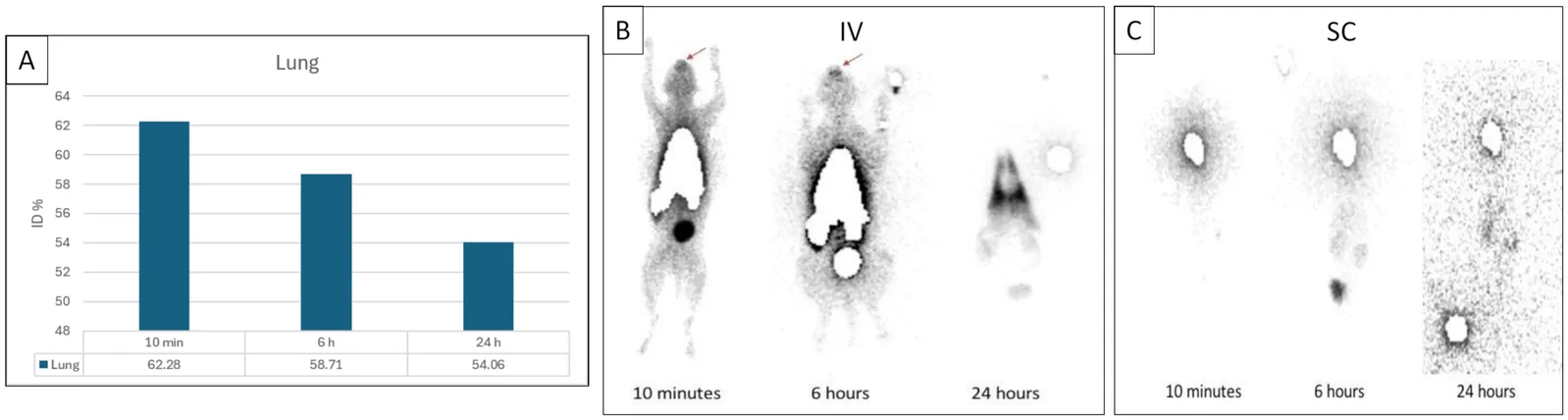
Distribution of ^99m^Tc labelled MSCs in cats with FCGS. Average percentage of injected dose (ID) observed in the lungs (**A**) and representative scintigraphic images following intravenous (IV) administration (**B**) and subcutaneous (SC) administration in the neck (**C**).

Subcutaneous injection of the ^99m^Tc labelled ePB-MSCs in the neck of the healthy cats led to an IRU in the liver for one cat and in the kidneys for another cat. In all three cats, there was IRU in the bladder, and a high IRU remained at the injection site. Finally, the subcutaneous injection of the ^99m^Tc labelled ePB-MSCs in the inguinal region of the healthy cats led to an IRU in the kidneys and the bladder in all three cats; the uptake increased until 6 h post-injection before decreasing. Again, a high IRU remained at the injection site throughout the study period (Figure 3).

Following IV injection of the ^99m^Tc labelled ePB-MSCs in the cephalic vein of the cats suffering from stomatitis, initial entrapment was seen in the lung. The accumulation in the lungs progressively decreased after injection. A low IRU was also observed in the liver, bladder, and kidneys. Additionally, IRU was observed in the rostral region of the mouth. At 6 h post-injection, a mean rostral-to-total head uptake ratio of 3.4 was observed (Table 4). The distribution of the radiolabelled ePB-MSCs to the head could also be observed on the scintigraphy images (hot spot) 6 h post-injection (Figure 3).

Following SC injection of the ^99m^Tc labelled ePB-MSCs in the neck of the cats suffering from FCGS, low IRU was observed in the kidneys and bladder in all four cats. Finally, a high IRU remained at the subcutaneous injection site throughout the study period.

## 4. Discussion

To the authors’ knowledge, this is the first study comparing the biodistribution pattern of ^99m^Tc labelled xenogeneic ePB-MSCs with free ^99m^Tc in cats free-^99m^Tc-labelled xenogeneic ePB-MSCs.

In the first and second studies, the mean labelling efficiency for ^99m^Tc labelled ePB-MSCs was 76% and 72%, respectively, which is consistent with previous studies conducted by our group [17].

Upon injection of free ^99m^Tc, it was observed that the radioactive uptake primarily occurred in the stomach, thyroid gland, and salivary glands, as reported by Mettler and Guiberteau [21]. This can be attributed to the binding of ^99m^Tc to plasma proteins and its rapid distribution to external fluids, leading to accumulation in the gastric mucosa, salivary glands, and thyroid gland. However, no radioactivity was observed in these organs after IV and SC injections of ^99m^Tc labelled ePB-MSCs, indicating the success and stability of the labelling technique used. Post-labelling viability varied between preparations and was as low as 53% in some samples, indicating that the labelling procedure or handling associated with the labelling process may have affected cell viability to a certain extent. In addition, variability in both administered cell dose and post-labelling viability may have contributed to interindividual differences in the biodistribution patterns observed in this study. If substantial release of free ^99m^Tc had occurred following cell death, accumulation would be expected in organs typically associated with free ^99m^Tc biodistribution, such as the stomach, thyroid gland, and salivary glands. As these uptake patterns were not observed following administration of labelled ePB-MSCs, widespread release of free radiotracer appears unlikely. However, the contribution of non-viable cells or released radiolabel to the detected scintigraphic signal cannot be completely excluded.

In contrast to a previous study conducted in dogs by our group [17], an initial pulmonary trapping of ePB-MSCs was observed following IV administration in healthy cats. Pulmonary first-pass entrapment is a well-recognized phenomenon following intravenous MSC administration and has been reported in several species, including mice, humans, and dogs. This phenomenon is generally attributed to the relatively large size of MSCs and their interactions with the pulmonary microvasculature [22,23]. Despite this initial sequestration, MSCs may subsequently redistribute and exert biological effects at distant sites of injury or inflammation [9]. The difference between the present study and our previous canine study may be related to species-specific factors, differences in injected cell numbers, or other methodological variables. In the canine study, initial pulmonary accumulation was only observed when higher cell doses were administered [17]. Similar pulmonary retention after intravenous MSC administration has also been reported in dogs using adipose-derived MSCs [22]. No clinical signs were associated with the pulmonary entrapment observed in the present study.

Following subcutaneous injection of ePB-MSCs, a significant radioactive uptake was observed at the injection site throughout the study period, which is consistent with the previously described biodistribution pattern in dogs [17].

In cats suffering from FCGS, a mean rostral-to-total head uptake ratio of 3.4 was observed following intravenous administration of ^99m^Tc-labelled ePB-MSCs. This should be interpreted as a descriptive observation given the small sample size and absence of statistical testing. The detection of radiotracer signal in the oral region of cats with FCGS, but not in healthy cats receiving the same treatment, is consistent with the localization of labelled ePB-MSCs, or their associated radiolabel, to inflamed oral tissues. Because no free ^99m^Tc control group was included in cats affected by FCGS, it cannot be excluded that local inflammation itself contributed to altered radiotracer accumulation. This is a preliminary study, and given that scintigraphy detects radioactivity rather than cell viability, these results remain descriptive and do not permit conclusions regarding functional engraftment. Imaging was limited to 24 h after administration due to the physical half-life of ^99m^Tc, preventing assessment of the potential later redistribution, persistence, or clearance of labelled cells.

Considerable interindividual variability was observed, including one animal showing a substantially higher rostral-to-total head uptake ratio than the other cats. Given the limited sample size, the source of this variability could not be determined. Potential contributing factors include biological variability between animals, differences in the severity or distribution of oral inflammation, and variability inherent to manual ROI delineation. As disease severity was not formally scored and image analysis was based on manually defined ROIs, these factors could not be further evaluated. Therefore, this observation should be interpreted cautiously.

There are several limitations to this exploratory pilot study. First, the number of included animals was limited, and no formal power calculation was performed, as no prior biodistribution data on the cats were available to support sample size estimation. Consequently, the findings should be interpreted descriptively and do not allow statistical inference or broad generalization. Second, due to the 6 h half-life of ^99m^Tc, imaging was limited to 24 h. Redistribution of MSCs beyond this timeframe, as described in other species, may not have been captured. Future studies may consider longer-lived radionuclides or multimodal labeling strategies to evaluate longer-term biodistribution. Third, although no abnormal clinical signs were observed during the short follow-up period, the long-term safety, immunogenicity, and tolerance of repeated administration of xenogeneic ePB-MSCs were not evaluated in the present study. Fourth, no free ^99m^Tc control group was included in the sample of cats affected by FCGS. Therefore, it cannot be definitively determined whether the increased radiotracer accumulation observed in the oral region reflects MSC-associated localization or differences in tracer biodistribution associated with inflammation. Future studies should include a free ^99m^Tc control group in the FCGS sample to clarify this aspect. Fifth, post-labelling viability varied between preparations and was reduced in some samples, which may have influenced the biodistribution results. Another limitation is that disease severity was not formally scored. Therefore, potential associations between the severity of oral inflammation and radiotracer accumulation could not be evaluated. Finally, image analysis was not performed in a blinded manner, which may have introduced observer bias. These limitations should be considered when interpreting the findings and warrant further investigation in larger controlled studies.

## 5. Conclusions

This study describes the biodistribution pattern of radiolabelled ePB-MSCs following intravenous and subcutaneous administration in healthy cats and in cats affected by FCGS based on scintigraphic imaging. A different biodistribution pattern was observed compared to free ^99m^Tc. Moreover, an increased radiotracer signal was detected in the rostral oral region of cats with FCGS 6 h following IV administration. By characterizing the biodistribution of radiolabelled ePB-MSCs, this exploratory study provides preliminary data that may support the design of future studies investigating MSC biodistribution and potential therapeutic applications in cats with FCGS. Additional studies are required to confirm these findings and to determine their clinical relevance.

## 6. Patents

J.H.S. (Jan H. Spaas)is an inventor of a pending patent covering the described immunomodulating technology, owned by GST (EP19162270.3). E.D., C.B., and J.H.S. (Jan H. Spaas) are the inventors of a patent covering the ^99m^Tc labelling of ePB-MSCs, owned by BIVMB (EP19200152.7). L.T., C.B., and J.H.S. (Jan H. Spaas)are the inventors of a patent covering the use of stem cells in the treatment of chronic gingivostomatitis, owned by BIVMB (EP21184499.8).

## Figures and Tables

**Table 1:** Injected ^99m^Tc activity and route of administration for control product in study 1.

Cat	Injection Route	Injected ^99m^Tc Activity (MBq)
1	IV	168.35
2		208.31
3		207.94
1	SC neck	162.06
2		182.41
3		187.22
1	SC inguinal region	209.42
2		227.18
3		207.57

MBq: megabecquerel, IV: intravenous, SC: subcutaneous.

**Table 2:** Route of administration, ^99m^Tc activity, labelling efficiency, number of cells, and cell viability of the labelled ePB-MSCs injected into each cat in study 1.

Cat	Injection Route	Injected ^99m^Tc Activity	Labelling Efficiency	Number of MSCs	Viability of MSCs
1	IV	269.73 MBq	74%	885,000	59%
2		268.99 MBq	68%	455,000	81%
3		304.88 MBq	83%	610,000	84%
1	SC neck	277.50 MBq	80%	510,000	83%
2		306.36 MBq	83%	720,000	76%
3		290.08 MBq	88%	650,000	70%
1	SC inguinal region	216.82 MBq	67%	430,000	53%
2		248.64 MBq	63%	500,000	76%
3		262.33 MBq	78%	475,000	62%
**MEAN**		**271.58 MBq**	**76%**	**581,667**	**72%**

MBq: megabecquerel, IV: intravenous, SC: subcutaneous.

**Table 3:** Route of administration, ^99m^Tc activity, labelling efficiency, number, and viability of the labelled ePB-MSCs injected into each cat in study 2.

Cat	Injection Route	Injected ^99m^Tc Activity	Labelling Efficiency	Number of MSCs	Viability of MSCs
1	IV	265.29 MBq	84%	310,000	87%
2		269.73 MBq	85%	230,000	80%
3		176.49 MBq	60%	285,000	88%
4		191.66 MBq	66%	260,000	77%
1	SC neck	190.18 MBq	89%	235,000	81%
2		186.11 MBq	51%	200,000	80%
3		257.15 MBq	78%	330,000	80%
4		250.49 MBq	66%	440,000	83%
**MEAN**		**223.11 MBq**	**72%**	**286,250**	**82%**

MBq: megabecquerel, IV: intravenous, SC: subcutaneous.

**Table 4:** Background-corrected mean counts per pixel observed in the rostral part of the head and in the total head of each cat and the ratio between both regions 6 h following intravenous injection of the radiolabelled ePB-MSCs in cats suffering from FCGS.

	CAT 1		CAT 2		CAT 3		CAT 4	
	Rostral Head	Total Head	Rostral Head	Total Head	Rostral Head	Total Head	Rostral Head	Total Head
6 h (counts/pixel)	5	4	9	1	7	3	22	7
BG-corrected ratio of rostral head to total head	1.1 FOLD		7.1 FOLD		2.2 FOLD		3.0 FOLD	
**MEAN BG-corrected ratio of rostral head to total head**	**3.4 FOLD**							

Ratios were calculated from background-corrected (unrounded) counts; therefore, values may differ slightly from ratios derived from the rounded counts presented in the table.

## Data Availability

The original contributions presented in this study are included in the article. Further inquiries can be directed to the corresponding author.

## Abbreviations

The following abbreviations are used in this manuscript:

ASC	Adipose-derived mesenchymal stem cell
CP	Control product
DMEM	Dulbecco’s Modified Eagle Medium
DMSO	Dimethylsulfoxide
ePB-MSC	Equine peripheral blood derived mesenchymal stem cell
FCGS	Feline chronic gingivostomatitis
FFV	Feline foamy virus
GMPs	Good manufacturing practices
ID	Injected dose
IRU	Increased radiopharmaceutical uptake
IV	Intravenous
IVP	Investigational veterinary product
MBq	Megabecquerel
MSC	Mesenchymal stem cell
ROI	Regions of interest
SC	Subcutaneous
^99m^Tc	^99m^Technetium
